# Measuring Quality of Public Hospitals in Croatia Using a Multi-Criteria Approach

**DOI:** 10.3390/ijerph18199984

**Published:** 2021-09-23

**Authors:** Nikola Kadoić, Diana Šimić, Jasna Mesarić, Nina Begičević Ređep

**Affiliations:** 1 Faculty of Organization and Informatics, University of Zagreb, Pavlinska Cesta 2, HR-42000 Varazdin, Croatia; diana.simic@foi.unizg.hr (D.Š.); nina.begicevic@foi.unizg.hr (N.B.R.); 2 Faculty of Health Sciences, Libertas International University, Trg J.F. Kennedy 6b, HR-10000 Zagreb, Croatia; jmesaric@libertas.hr

**Keywords:** analytic hierarchy process, group decision making, composite indicator, healthcare policy, hospital, performance indicator

## Abstract

Quality of public hospital services presents one of the most important aspects of public health in general. A significant number of health services are delivered due to public hospitals. Under the World Bank program “Improving Quality and Efficiency of Health Services: Program for Results”, the competent bodies in Croatia aimed to identify the top 40% best-performing public acute hospitals in Croatia, based on a clinical audit in the preceding 12 months. This paper presents how this goal was achieved, using a multi-criteria decision-making (MCDM) approach. A MCDM approach was selected due to the multidimensionality and complexity of healthcare performance and service quality. We aimed to develop a methodology for ranking top-performing hospitals at the national level. We chose the composite indicator methodology, combined with the analytic hierarchy process (AHP) as a tool for determining weights for aggregation of individual indicators. The study looked at three clinical entities: acute myocardial infarction, cerebrovascular insult, and antimicrobial prophylaxis in colorectal surgery. Indicators for each entity were evidence-based, following the national guidelines, but limited by availability of data. The clinical audit and databases of competent administrative bodies were used as sources of data. The problem investigated in this paper has a significant impact at the strategic (national) level. Even though the AHP has already been applied in the public health domain, to the best of our knowledge, this is the first application of the AHP in combination with composite indicators for hospital ranking at a national level. The AHP enabled participation of experts from the audited hospitals in the assessment of indicator weights. Results show that composite indicators can be successfully implemented for acute hospital evaluation using the AHP methodology: (1) the AHP supported a flexible structuring of the problem; (2) the resulting complexity of pairwise comparisons was appropriate for the experts (consistency ratios were under 0.1); (3) using the AHP approach enabled a successful aggregation of different opinions into group priorities; (4) the developed methodology was robust and enabled identifying the top 40% ranking best-performing public acute hospitals in Croatia combining 20 criteria within three entities, based on input from 36 clinical experts. The proposed methodology can be useful to other researchers for assessment of healthcare quality at the strategic level.

## 1. Introduction

### 1.1. The Background: A World Bank Program

Under the World Bank program “Improving Quality and Efficiency of Health Services: Program for Results”, the competent bodies (the Ministry of Health of the Republic of Croatia, the Croatian Health Insurance Fund, and the Agency for Quality and Accreditation in Health and Social Care) had a goal to identify the top 40% best-performing acute hospitals in the Republic of Croatia, based on the technical (clinical) audit in the preceding 12 months. To achieve this goal, the Agency for Quality and Accreditation in Health and Social Care (AQAH) defined a protocol for a technical/clinical audit and conducted an audit in 28 Croatian acute hospitals. The audit was carried out with respect to three clinical entities: acute myocardial infarction (AMI), cerebrovascular insult (CVI) and antimicrobial prophylaxis in colorectal surgery (APC). During the audit, the AQAH collected a wide range of data on compliance of clinical practices with the national guidelines [1,2,3], patient safety indicators, and administrative data. Constructing an indicator for ranking that would be evidence-based, scientifically grounded, and acceptable to the audited hospitals was a challenge. We decided to combine a methodology for constructing a composite index with group multi-criteria decision-making for determining weights of individual indicators, aiming to involve the audited hospitals in a participatory decision-making process.

Foundations for evidence-based individual indicators used in this paper were laid down in 2003, when the World Health Organization (WHO) Regional Office for Europe launched a project aiming to develop and disseminate a flexible and comprehensive tool for the assessment of hospital performance, referred to as the performance assessment tool for quality improvement in hospitals (PATH). The project aimed at supporting hospitals in assessing their performance, analyzing their results, and translating them into actions for improvement, by providing hospitals with tools for performance assessment, and by enabling collegial support and networking among participating hospitals [4]. The first phase of the PATH project was piloted in eight countries to refine its framework before further expansion. In 2008 Croatia joined the project with 22 participating hospitals [5]. Three individual indicators of patient safety developed within the PATH project were used in our research: Patient-based AMI 30 day in-hospital mortality rate, Patient-based CVI 30 day in-hospital mortality rate, and prophylactic antibiotic use.

The development of performance indicators for monitoring, assessing, and managing health systems to achieve effectiveness, equity, efficiency, and quality is a subject of interest in many countries and international organizations [6]. Arah et al. [6] discuss that it is often not very clear just what the underlying concepts might be, or how effectiveness is conceptualized and measured. Therefore, they explore, individually, the conceptual bases, effectiveness and related indicators, as well as quality improvement dynamics of performance frameworks of the United Kingdom, Canada, Australia, the United States of America, the World Health Organization, and the Organization for Economic Co-operation and Development. At the level of provider institutions they identify use of “accreditation and certification; public disclosure of performance, benchmarking, and comparisons using standardizes indicators” as tools for extrinsic regulation. In all analyzed frameworks, they note an implicit or explicit association between the effectiveness and quality. Healthcare quality has been a focus of interest for a long time. Indeed, a review paper dealing with description and evaluation of current methods for assessing quality of healthcare was published as early as 1973 [7]. In 1988 Donabedian [8] explores in depth the concept of healthcare quality, and defines three types of indicators that can be used for its assessment—structure, process, and outcome indicators. The Donabedian’s conceptual model is still a standard framework for evaluating quality of healthcare.

### 1.2. A Multi-Criteria Approach for Measuring Quality

Composite performance measures are increasingly being used in healthcare systems, because they can present a “big picture” of the system. Jacobs et al. [9] assess robustness of hospital ranks based on composite performance measures and discuss possible issues in the construction of composite indicators. They describe how variability in underlying data and the methodological decisions can have a large impact on composite scores. In their analysis, ranks of some hospitals can change by almost a half of the league table as a result of subtle changes in data or methodology. Saisana et al. [10] propose using uncertainty and sensitivity analyses to gain useful insights during a process of building composite indicators in the context of policy development and country rankings. They also discuss to what extent uncertainty and sensitivity analyses may contribute to transparency or make policy inference more defensible. Reeves et al. [11] pursue a similar goal. They work on creating a composite indicator as a quality measure combining multiple indicators of clinical quality. The authors compare five different methods of aggregation: All-or-None, 70% Standard, Overall Percentage, Indicator Average, and Patient Average. The results show variations depending on the method of aggregation used. Different methods are suited to different types of applications. Advantages and disadvantages of various methods are described and discussed in [12]. Shwartz et al. [13] also discuss composite measures of healthcare providers. They analyze the necessary trade-offs and knowledge gaps, and provide recommendations for selecting an approach to developing composite indicators.

The Analytic Hierarchical Process (AHP) has been applied in different fields: management, resource allocation, distribution, education, healthcare, industry, government and other fields. In most cases, it is applied for making strategic decisions, but also there are applications at the tactical and operative levels. It is considered one of the most popular multi-criteria decision-making methods [14]. The reason the AHP is so popular is that it has many advantages. For instance, with the AHP discussions about a decision-making problem are much more structured and better organized; only two elements are compared at the same time—which simplifies judgments; decision-makers have more confidence in the result because they have participated in the procedure; the AHP combines both qualitative and quantitative parameters; there is a mechanism for resolving inconsistencies; redundancy in providing judgments decreases probability of failures in the process; there is a software support for the method [15,16].

Use of the AHP in healthcare can be traced to 1990s [17]. More recent uses include selection of infectious medical waste disposal companies [18], ranking the macro-level critical success factors of electronic medical record adoption [19], health technology assessment [20], calculation of quality-adjusted life years [21], renewal of technology for healthcare equipment [22] and many others. Comprehensive literature review studies on applications of the AHP in medicine and healthcare were carried out by Liberatore and Nydick [23], Ho [24], Schmidt et al. [25], and Ho and Ma [14].

### 1.3. Measuring Quality of Hospitals in Croatia

To determine the best-performing hospitals with respect to the chosen clinical entities, it was necessary to identify criteria of performance on each of the three entities and a method of aggregation. Following the selection of the criteria and the aggregation method, it was necessary to determine relative importance of the criteria, i.e., their weights or priorities. For that purpose the AHP, a multi-criteria decision-making method, was used.

The findings discussed in this paper are part of a broader project aimed at identifying the top-performing hospitals in the Republic of Croatia.

**Figure 1:**
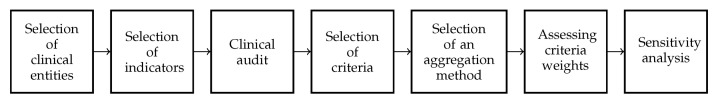
The conceptual model of the process for identifying the top 40% best-performing public acute hospitals in Croatia.

The conceptual framework of the project is presented in Figure 1.

**Selection of clinical entities**—was based on national priorities and national clinical guidelines, aiming to assess quality and level of implementation of national guidelines in the clinical practice, as well as efficiency.

**Selection of indicators**—implied choosing evidence-based indicators of hospital healthcare quality and patient safety, as well as indicators of efficiency, and identifying sources of data for computing the indicators. In addition to the clinical audit, data were also collected from national health information systems of the AQAH and the Croatian Health Insurance Fund (CHIF).

**Clinical audit**—comprised independent review of medical documentation (a random sample of 50 medical histories per hospital per clinical entity) carried out by the AQAH staff. Data for computing indicators that were not available from national health information systems at AQAH and CHIF were collected during the audit.

**Selection of criteria**—that were used in the composite indicators was based on availability and quality of data from the national health information systems and the clinical audit. We took a pragmatic approach, excluding indicators when discrepancies in data collection procedures between hospitals rendered the results incomparable.

**Selection of an aggregation method**—also involved selection of a normalization or scaling method. We chose the linear additive aggregation, because it is easiest to interpret contribution of individual indicators to the composite indicator. Scaling was linear with truncation of extreme values. For each indicator scaling was selected such that ranges of normalized values across the audited hospitals were similar.

**Assessing criteria weights**—was done using the AHP. Criteria for pairwise comparisons were defined taking into account selected scaling of the indicators. Group priorities obtained through the AHP were used as weights in linear aggregation.

**Sensitivity analysis**—was done by Monte Carlo simulation with 100,000 replications drawing weights from uniform distribution on an interval of ±15% around the weights

In this paper, we focus on the assessment of criteria weights, which was based on the AHP, and the sensitivity analysis. Our objective is to demonstrate how the AHP can be used for group decision-making in the process of designing a composite indicator of hospital performance. We provide information on data collection, and explain the AHP method and the sensitivity analysis in the next section. Results of the group decision making with the AHP, and the sensitivity analysis are presented next, followed by a discussion and conclusions.

The research goals of this paper are:

1. To establish a methodology for ranking the top-performing hospitals at the national level that will enable participation of clinical experts, and aggregation of their, possibly conflicting, opinions,
2. To apply the methodology in the case of Croatian public acute hospitals.

### 1.4. Contributions

Contributions of this research include:

1. Even though the AHP was already applied to some problems in the public health domain, this is, to the best of our knowledge, the first application of the AHP in combination with the composite indicator methodology for ranking hospitals at the national level.
2. Experts and representatives of all the audited hospitals participated in the decision-making process. Since the experts analyzed the problem from their own perspectives, using the AHP approach enabled a successful aggregation of different opinions into group priorities. Participatory design of the composite indicators contributed to building of trust and acceptance of the ranking results.
3. Results show that designing composite indicators for acute hospital evaluation can be successfully implemented using the AHP methodology. The presented case can be useful to other researchers assessing healthcare quality at the strategic level. The problem investigated in this paper has a significant impact at the strategic (national) level.

## 2. Materials and Methods

Hospital quality and performance are complex multidimensional concepts, and any approach to hospital ranking must take into account multiple criteria. There is a vast choice of MCDM methods that can be used for decision-making, clustering and prioritization. Hospital ranking is a problem of prioritization, and the choice of MCDM methods that can be used include the AHP, the Analytic Network Process (ANP), Electre, Promethee, Topsis, Vikor, Dex, and many others [15]. Choice of a multi-criteria method can be based on several criteria, e.g.,

**Method acceptance.** Among all MCDM methods, the AHP is the most often used in terms of both frequency and application domains. It is almost impossible to find a domain in which the method has not been applied. There are already some applications of the method in the area of public health (see Section 1.2).

**Support for the group decision making.** Most MCDM methods do not support sophisticated group decision making. Usually, group decision-making is implemented naively: (1) the priorities are calculated individually, and then aggregated using the arithmetic mean or (2) they require that the members of group agree on value that needs to be input in the method. In the AHP, the instrument for aggregating individual judgments respects individual opinions (without a need to achieve a compromise during the data collection procedure) and it is not naive—it is implemented as the geometric mean at the level of single pairwise comparisons. Group decision making is best supported in the AHP.

**Criteria prioritization procedure.** In most MCDM methods the prioritization procedure takes some form of rating (direct assessment): e.g., an expert assesses importance of a criterion by allocating a sum of 100% over all criteria. In the AHP and the ANP criteria are compared pairwise, and experts provide judgments on each criterion several times before reaching final criteria priorities. It is also possible to evaluate consistency of experts’ assessments across all criteria.

**Dependencies between the criteria.** The ANP was specifically designed to model dependencies between criteria. Most other MCDM methods, including the AHP, do not take these dependencies into account. Dependencies between criteria in our model were relatively low.

**Method complexity.** When two methods meat all requirements, it is prudent to choose a simpler method. The AHP is less complex than the ANP (the number of inputs for the AHP is lower, the data collection procedure is shorter, and it is easier for experts to understand the required inputs).

Both the AHP and the ANP satisfy the first three criteria. An advantage of the ANP is that it provides a mechanism to incorporate dependencies between the criteria, while the AHP is simpler in terms of number of inputs, data collection, computation and interpretation. Since dependencies between the criteria in our case were relatively low, the AHP was our method of choice.

**Figure 2:**
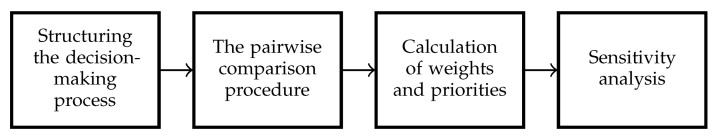
The analytic hierarchy process workflow.

**Figure 3:**
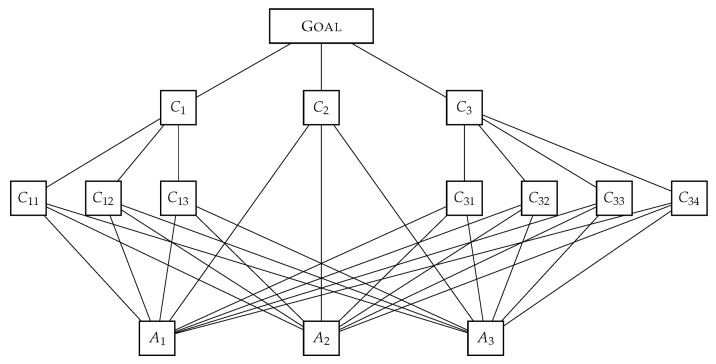
An example of a structure with three criteria ($C_{1}$ to $C_{3}$), seven subcriteria ($C_{11}$ to $C_{34}$), and three alternatives ($A_{1}$ to $A_{3}$).

The AHP is one of the best known and the most often used multi-criteria decision-making methods. The author of the AHP is Prof Thomas Saaty. The overall AHP process consists of four steps, shown as a workflow in Figure 2 [26,27]:

**Structuring the decision-making problem.** In the AHP, the problem is structured as a hierarchy. At the top of the hierarchy, there is a decision-making goal. The goal depends on criteria, which can be decomposed into subcriteria (i.e., further levels). Finally, at the last level, there are alternatives. Figure 3 presents a structure that consists of one goal, three criteria, seven subcriteria, and three alternatives. Of course, it is possible that in some decision-making context, we face truncated hierarchy, a hierarchy in which criteria or alternatives are missing. Mu et al. [28] provide an example of a case with missing criteria. The problem analyzed in this paper is an example of a case when the alternatives are not known (actually, the hospitals are the alternatives, but they will be evaluated using composite indicators, the AHP is used only for determining the criteria weights). Methods that can be useful in terms of structuring phase of the AHP are [29]:

1. interviews with experts in the problem domain,
2. literature review (searching for examples of relevant decision-making problems in scientific and/or professional literature),
3. brainstorming and other creativity techniques (for generating new alternatives),
4. Delphi technique [30] can be used when agreeing on the hierarchy in terms of its completeness and structure,
5. top-down and bottom-up approaches in creating a hierarchy (after its elements are identified),
6. The Problem formulation, Objectives, Alternatives, Consequences, Trade-offs, Uncertainties, Risk attitude, and Linked decisions (PrOACT) approach in decision-making problem decomposition [31],
7. thinking about the problem, reasoning, reflexing, synthesis.

**The pairwise comparison procedure.** Here, elements at a certain level of the hierarchy are pairwise compared with respect to an element at the higher level in the hierarchy. For example, for the structure in Figure 3, criteria $C_{1},C_{2}$, and $C_{3}$ will be pairwise compared with respect to the goal; subcriteria $C_{11},C_{12}$, and $C_{13}$ will be pairwise compared with respect to Criterion $C_{1}$; subcriteria $C_{31},C_{32},C_{33}$, and $C_{34}$ will be pairwise compared with respect to the Criterion $C_{3}$; and finally, alternatives $A_{1},A_{2}$, and $A_{3}$ will be pairwise compared in respect to subcriteria $C_{11},C_{12},C_{13},C_{31},C_{32},C_{33}$, and $C_{34}$ and Criterion $C_{2}$.

**Calculation of weights and priorities.** Each set of pairwise comparisons from the previous step generates a comparison matrix. In the example from Figure 3, 11 pairwise comparison matrices will be created. For each pairwise comparison matrix, attention must be paid to the consistency ratio. Additionally, in the case of group decision making, it is important to ensure that the group pairwise comparison matrix is consistent, too. After criteria weights, subcriteria weights and alternatives’ priorities with respect to the subcriteria and Criterion 2 are calculated, they are aggregated into the final priorities using simple additive weighting (SAW).

**Sensitivity analysis.** In the last step, analysis of the sensitivity of the outputs (alternatives’ priorities) to ±5% change of inputs (criteria weights) must be done before reaching the final decision or changing the approach or the method.

In the rest of this section, we provide description of each of the steps in the AHP workflow, and provide details on how they were performed in our research.

### 2.1. Structuring the Decision-Making Problem

Three clinical entities were selected for the audit: acute myocardial infarction (AMI), cerebrovascular insult (CVI) and antimicrobial prophylaxis in colorectal surgery (APC). AMI and CVI were chosen, because diseases of circulatory system are the main cause of mortality in Croatia (42% of deaths in 2019 [32]) and the European Union (37% deaths in 2017 [33]). On the other hand, antimicrobial resistance is a significant global healthcare problem [33]. APC was chosen because the misuse and overuse of antibiotics contributes to the development of antimicrobial resistance and increases the risk of hospital infections. Additionally, it was important that national guidelines, a common reference for all audited hospitals, exist for all three chosen entities [1,2,3].

Data for comparing public acute hospitals in Croatia came from three sources:

1. The audit procedure in the hospitals,
2. Reports of the Agency for Quality and Accreditation in Health and Social Care (AQAH), and
3. Information system of the Croatian Health Insurance Fund (CHIF).

The data comprised patient safety indicators reported by the AQAH [34], indicators of compliance with national clinical guidelines based on data collected during the audit [1,2,3], and efficiency and effectiveness indicators based on invoice database of the CHIF. They were grouped into indicators related to AMI, CVI, and APC.

For each entity, the choice of indicators was also based on availability of data for all hospitals, and comparability of procedures for data collection among the hospitals. Final indicators for AMI, CVI, and APC are presented in Table 1.

**Figure 4:**
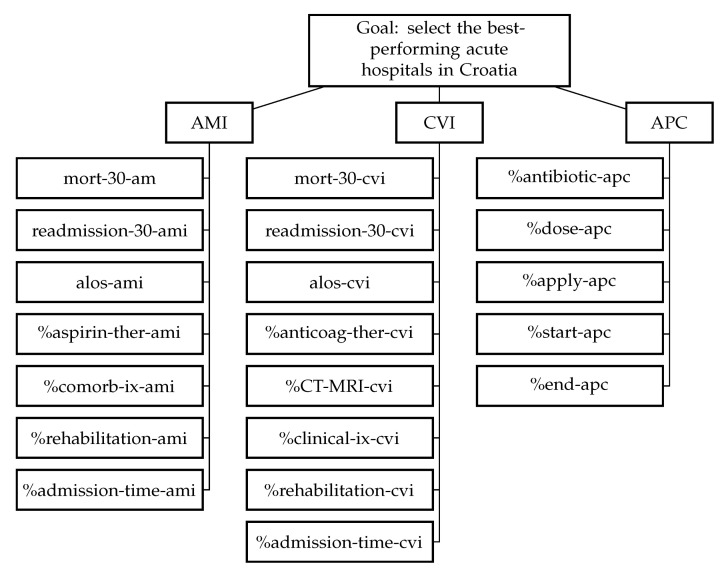
A hierarchical tree for selecting the best-performing acute hospitals in Croatia AMI = acute myocardial infarction; CVI = cerebrovascular insult; APC = antimicrobial prophylaxis in colorectal surgery, criteria below the entities are labeled using abbreviations from Table 1.

The hierarchical structure of the problem, using abbreviations from Table 1 is presented in Figure 4. At the top of the hierarchy is the decision-making goal: identification of the best-performing hospitals in Croatia. At the lower level, there are entities as the main criteria. Finally, at the second level, there are the subcriteria, criteria derived from the indicators presented in Table 1.

There were 28 public acute hospitals included in the audit. All audited hospitals have cardiology and surgery departments (sources of AMI and APC data). Only 25 audited hospitals have a neurology department (source of CVI data). Therefore, we could not create a single ranking combining all three entities, and a separate ranking was created for each entity.

### 2.2. The Pairwise Comparison Procedure

#### 2.2.1. The Saaty’s Scale

The AHP method is based on a pairwise comparison procedure, which uses the Saaty scale [35] (Table 2).

To rank objects using the AHP, we first select criteria to be used for comparison. Both quantitative and qualitative criteria can be used. For a qualitative criterion, a lower hierarchy level is created under it, with all its possible values, usually called alternatives. The pairwise comparison procedure can be used for both estimating criteria weights and calculating the alternatives’ priorities with respect to a criterion. There are several methods for estimating priorities (or weights) given a pairwise comparison matrix.

For example, one could ask experts to provide their assessments on what is more important and by how much—decreasing a readmission rate by 5% or decreasing an average length of hospital stay by 1 day. If an expert decided that a pairwise comparison between these criteria was 3 on the Saaty’s scale, it would mean that it is moderately more important to decrease a readmission rate by 5% than to decrease an average length of hospital stay by 1 day.

#### 2.2.2. The Axioms of the AHP

The AHP method is based on four axioms [36]. Let $A_{i},i=1,\ldots ,n$ be alternatives to be compared with respect to a criterion *C*. Let $P_{C}\left(A_{i},A_{j}\right)$ be a mapping that assigns to each pair of alternatives their relative importance with respect to a criterion *C*. $P_{C}\left(A_{i},A_{j}\right)>1$ means that $A_{i}$ is more important than $A_{j}$, and the strength of the dominance is interpreted according to Table 2.

**Axiom** **1.** **The reciprocal axiom.** For all $A_{i},A_{j}$

$$P_{C}\left(A_{i},A_{j}\right)=\frac{1}{P_{C}\left(A_{j},A_{i}\right)}.$$

For example, if an expert decided that it was moderately more important to decrease a readmission rate by 5% than to decrease an average length of hospital stay by 1 day (3 on a Saaty scale), then, by the reciprocal axiom, it is moderately less important to decrease an average length of hospital stay by 1 day then to decrease a readmission rate by 5% (1/3 on the Saaty scale). Thus, for each pair of criteria or alternatives, we need only obtain a pairwise comparison in one direction, and the other direction follows from the reciprocal axiom.

**Definition** **1.** *Let $S=\{A_{i}\}$ be a finite partially ordered set. We say that $A_{i}$ covers $A_{j}$ if $A_{i}>A_{j}$ and $A_{i}\geq A_{k}>A_{j}\Rightarrow A_{i}=A_{k}$. $A_{i}^{-}$ is defined as $A_{i}^{-}=\left\{A_{j}|A_{i}\mathrm{covers}A_{j}\right\}$ and $A_{i}^{+}=\left\{A_{j}|A_{j}\mathrm{covers}A_{i}\right\}$. S is a hierarchy if it satisfies the following conditions:*

*1.*  *There is a single largest element A∈S.*
*2.*  *There is a partition of S, $P\left(S\right)=L_{i},i=1,\ldots ,k$ into sets called levels, such that*
  *(a)*  *$L_{1}=\left\{A\right\}$.*
  *(b)*  *$x\in L_{i}\Rightarrow x^{-}\subseteq L_{i+1}\mathrm{for}i=1,2,\ldots ,k-1$.*
  *(c)*  *$x\in L_{i}\Rightarrow x^{+}\subseteq L_{i-1}\mathrm{for}i=2,3,\ldots ,k$.*

*For any positive real number ρ∈R, ρ≥1 a nonempty set $x^{-}\subseteq L_{i+1}$ is ρ-homogenous with respect to $x\in L_{i}$ if for any pair of elements, $A_{i},A_{j}\in x^{-}$, $\frac{1}{\rho }\leq P_{C}\left(A_{i},A_{j}\right)\leq \rho$.*

We can take as an the example the structure in Figure 3, with a partial order relation between the criteria/alternatives *X* and *Y* defined in this way: X>Y if *X* is above *Y*, and we can trace a downward line from *X* to *Y* (with possible intermediaries). Thus, $C_{1}$ is greater than any of $C_{11},C_{12},C_{13},A_{1},A_{2},A_{3}$, but it is not greater than $GOAL,C_{2},C_{3}$, nor $C_{21},C_{22},C_{23},C_{24}$. In this example,

$$S=\{GOAL,C_{1},C_{2},C_{3},C_{11},C_{12},C_{13},C_{21},C_{22},C_{23},C_{24},A_{1},A_{2},A_{3}\}.$$

The single largest element of *S* is GOAL (Definition 1, rule 1). Levels are (Definition 1, rule 2):

1. $L_{1}=\left\{GOAL\right\}$
2. $L_{2}=\left\{C_{1},C_{2},C_{3}\right\}$
3. $L_{3}=\left\{C_{11},C_{12},C_{13},C_{21},C_{22},C_{23},C_{24}\right\}$
4. $L_{4}=\left\{A_{1},A_{2},A_{3}\right\}$

$C_{1}$ covers $C_{11},C_{12}$, and $C_{13}$, because, if we take any of these criteria *X*, the only element Y∈S such that $C_{1}\geq Y>X$ is the $C_{1}$ itself. On the other hand, GOAL does not cover $C_{11}$, because $GOAL\geq C_{1}>C_{11}$, and $GOAL\neq C_{1}$. GOAL does cover $C_{1},C_{2}$, and $C_{3}$. According to rule 2(b) for $C_{1}\in L_{2}$, $C_{1}^{-}=\left\{C_{11},C_{12},C_{13}\right\}\subseteq L_{3}$. According to rule 2(c) $C_{1}\in L_{2}$, and $C_{1}^{+}=\left\{GOAL\right\}\subseteq L_{1}$. On the other hand, for $C_{2}\in L_{2}$, $C_{2}^{-}=\left\{A_{1},A_{2},A_{3}\right\}⊈L_{3}$. That means that structure in Figure 3 is not a hierarchy according to Definition 1, and we need to insert a criterion $C_{21}$ at level $L_{3}$ between $C_{2}$ at the second level and the alternatives at the fourth level, in order to transform it into a hierarchy satisfying the Definition 1.

For any criterion *X*, $X^{-}$ is a set of criteria that will be pairwise compared with respect to *X*. If $X^{-}$ is ρ−homogeneous with respect to *X*, then the largest ratio of importance between any pair of criteria/alternatives from $X^{-}$ with respect to *X* will be at most ρ. Since Saaty’s scale can only take integer values 1 to 9 and their reciprocals, any set of criteria/alternatives that enter into pairwise comparisons must be 9-homogeneous. That is why we need the homogeneity axiom.

**Axiom** **2.** **The homogeneity axiom.** Given a hierarchy P(S) with *k* levels, x∈S, and $x\in L_{i}$, than $x^{-}\subseteq L_{i+1}$ is ρ-homogeneous for i=1,2,…,k−1.

Saaty [36] argues that human mind cannot compare very different elements with adequate precision. That is why he proposes to group similar elements in clusters of comparable sizes, and to introduce new hierarchy levels to achieve this goal. The partition P defines a structure of a multi-criteria decision problem, and the homogeneity axiom requires that the structure be such that experts doing the pairwise comparisons can provide reasonably accurate estimates of relative importance of criteria and alternatives. In a hierarchy, elements of $x^{-}$ are compared pairwise with respect to *x* to obtain a local derived scale, or local priorities.

**Definition** **2.** *A set A is outer dependent on a set C if a fundamental scale (Table 2) can be defined on A with respect to every c∈C. If A is outer dependent on C, we say that elements of A are* ***inner dependent*** *with respect to c∈C if there is an A∈A, such that A is outer dependent on {A}.*

**Axiom** **3.** **The dependency axiom**. Let P(S) be a hierarchy with levels $L_{1},L_{2},\ldots ,L_{k}$. For each $L_{i},i=1,2,\ldots ,k-1$:

1. $L_{i+1}$ is outer dependent on $L_{i}$.
2. $L_{i}$ is not outer dependent on $L_{i+1}$.
3. $L_{i+1}$ is not inner dependent with respect to any $A\in L_{i}$.

The dependency axiom establishes dependencies within a hierarchy such that a lower level depends on the adjacent higher level.

Let us assume that a decision-maker has an intuitive ranking of a finite set of alternatives A with respect to prior knowledge of criteria C. We call these beliefs about the rank of alternatives expectations.

**Axiom** **4.** **The expectations axiom**. There is an *i* such that $C\subset S\setminus L_{i}$, $A=L_{i}$ (completeness).

The expectations axiom reflects the idea that an outcome can only reflect expectations when the latter are well represented in the hierarchy.

#### 2.2.3. The Comparison Matrix

Next, we describe the pairwise comparison procedure. Let us say that we have *n* alternatives $A_{1},\ldots ,A_{n}$ that we need to prioritize (estimate weights/priorities) with respect to some criterion *C*. The procedure is as follows:

Create a square n×n matrix $M=[m_{ij}]$ where $m_{ij}$ are pairwise comparisons of alternatives $A_{i}$ and $A_{j}$ with respect to criterion *C* using the Saaty scale (Table 2):

1. $m_{ii}=1,i=1,2,\ldots ,n$.
2. $m_{ij}=P_{C}\left(A_{i},A_{j}\right)$, i≠j, i,j=1,…,n.

From the reciprocal axiom we can derive that $m_{ji}=\frac{1}{m_{ij}}$. When comparing alternatives $A_{i}$ and $A_{j}$ the question that the decision-maker should answer is “Which alternative, $A_{i}$ or $A_{j}$, is more important with respect to the context, and by how much on the Saaty scale.”

For example, with n=3, one can say that alternative $A_{2}$ is moderately more important than alternative $A_{1}$. This means that $m_{21}=3$, and $m_{12}=\frac{1}{3}$. In general, a Saaty value higher than 1 is inserted in the row corresponding to the alternative that dominates over another, and the reciprocal value is inserted in the symmetric position. Similarly, if $A_{1}$ dominates over $A_{3}$ by 2 on the Saaty scale, then $m_{13}=2$, and $m_{31}=\frac{1}{2}$. Finally, if $A_{2}$ dominates over $A_{3}$ by 5 on the Saaty scale, then $m_{23}=5$, and $m_{32}=\frac{1}{5}$. The pairwise comparison matrix for this example is:

(1) $$M=\begin{array}{cc} \begin{array}{ccc} A_{1} & A_{2} & A_{3} \end{array} & \\ \left[\begin{array}{ccc} 1 & \frac{1}{3} & 2\\ 3 & 1 & 5\\ \frac{1}{2} & \frac{1}{5} & 1 \end{array}\right] & \begin{array}{c} A_{1}\\ A_{2}\\ A_{3} \end{array} \end{array}$$

If only the AHP were used for prioritization of the hospitals, in addition to doing pairwise comparisons between the criteria, the experts would also have to do pairwise comparisons between hospitals (as alternatives) in respect to every criterion. For the CVI, which had eight criteria for the 28 hospitals, that would mean $8\times \frac{28\;\times \;27}{2}=3024$ additional pairwise comparisons. Instead, we calculated a composite indicator for each entity as a weighted sum of normalized individual indicators, using the criteria weights obtained by the APH.

Since we used the AHP to estimate indicator weights, we had to introduce the scale of indicators in the pairwise comparison. During the pairwise comparisons, experts compared criteria defined as a specified difference in the value of an indicator, e.g., a decrease in average hospital stay by one day. This was important, because these criteria also defined the scaling factors later used for normalization of individual indicators. The number of pairwise comparisons for an entity with *k* indicators is $\frac{k\cdot (k-1)}{2}$. Thus, there were 21 comparisons for the AMI, 28 for the CVI, and only 10 for the APC.

#### 2.2.4. Group Decision Making Using the AHP

We have taken advantage of the AHP method’s ability to facilitate collaborative decision-making. Experts independently provided pairwise comparisons, which were subsequently aggregated into group pairwise comparisons. This aggregation is usually done in one of the following two ways:

1. Different experts provide pairwise comparisons on disjoint sets of criteria or alternatives. An example of this case can be found in a paper by Mu and Stern [37].
2. A group of *l* experts compares the same criteria or alternatives. An expert *k* provides a pairwise comparison matrix $M^{\left(k\right)}=\left[m_{ij}^{\left(k\right)}\right]$. Aggregated group pairwise comparison matrix $M=[m_{ij}]$ is computed from individual matrices using the geometric mean $m_{ij}=\sqrt[{l}]{\prod_{k=1}^{l}m_{ij}^{\left(k\right)}}$.

Here is an example of group decision making using geometric mean aggregation:

$$M^{\left(1\right)}=\left[\begin{array}{ccc} 1 & \frac{1}{3} & 2\\ 3 & 1 & 5\\ \frac{1}{2} & \frac{1}{5} & 1 \end{array}\right]\;\;M^{\left(2\right)}=\left[\begin{array}{ccc} 1 & \frac{1}{2} & 3\\ 2 & 1 & 5\\ \frac{1}{3} & \frac{1}{5} & 1 \end{array}\right]\;\;M^{\left(3\right)}=\left[\begin{array}{ccc} 1 & \frac{1}{3} & 2\\ 3 & 1 & 5\\ \frac{1}{2} & \frac{1}{5} & 1 \end{array}\right]\;\;M=\left[\begin{array}{ccc} 1 & \frac{1}{\sqrt[{3}]{18}} & \sqrt[{3}]{12}\\ \sqrt[{3}]{18} & 1 & 5\\ \frac{1}{\sqrt[{3}]{12}} & \frac{1}{5} & 1 \end{array}\right]$$

To promote a participatory decision-making, one expert per entity from each audited hospital was invited to participate in the pairwise comparisons process. Experts’ assessments of the importance of criteria represented the perspectives of their respective hospitals. For each entity, a collaborative focus group meeting was organized at the Faculty of organization and informatics. At the meetings, context of the World Bank project was explained, relevant indicators were described and discussed until common understanding was reached. Experts actively participated in the focus group meeting, as official representatives of their hospitals, without distractions from everyday duties. The focus group sizes were nine for the AMI, 16 for the CVI, and 11 for the APC.

Measuring of the group agreement/disagreement was not important for the purpose of this project. It was clear from the very beginning that we will witness both agreements and disagreements. The goal was to reach a compromise, and it was agreed that the compromise will be achieved using group decision making, in which all the experts will have an equal importance.

### 2.3. Calculation of Weights and Priorities

When a pairwise comparison matrix is created, there are several possible approaches to calculating the priorities of alternatives $A_{1},A_{2},\ldots ,A_{n}$. The optimal method is to compute the largest eigenvalue and the corresponding eigenvector. Elements of the reciprocal matrix M are strictly positive $m_{ij}>0$, thus Perron Frobenius theorem guarantees that it has a unique largest real eigenvalue and that the corresponding eigenvector can be chosen to have strictly positive components. Since eigenvectors are scale invariant, the eigenvector is usually normalized to have the sum of elements equal 1. If using manual calculations, there are several approaches to approximating the largest eigenvalue and the corresponding eigenvector. Here, we present one of them:

1. In this procedure, the first step is to normalize each column of the comparison matrix to the sum of 1. Let $e={\left[\begin{array}{ccc} 1 & \cdots & 1 \end{array}\right]}^{T}$ be a column vector of length *n*. Column sums of matrix M are computed as $s=e^{T}\cdot M$. Next, the comparison matrix is normalized by column sums: $\tilde{M}=M\cdot {\left[\mathrm{diag}\left(s\right)\right]}^{-1}$ where diag(s) is a diagonal n×n matrix with the elements of vector s on the diagonal.
2. The second step is to estimate priorities *p* as row averages of the normalized matrix $\tilde{M}$:
  $$p=\frac{1}{n}\tilde{M}\cdot e.$$

For the comparison matrix (1):

$$\;\begin{array}{cccc} M=\left[\begin{array}{ccc} 1 & \frac{1}{3} & 2\\ 3 & 1 & 5\\ \frac{1}{2} & \frac{1}{5} & 1 \end{array}\right] & s=\left[\begin{array}{ccc} \frac{9}{2} & \frac{23}{15} & 8 \end{array}\right] & \tilde{M}=\left[\begin{array}{ccc} \frac{2}{9} & \frac{5}{23} & \frac{1}{4}\\ \frac{2}{3} & \frac{15}{23} & \frac{5}{8}\\ \frac{1}{9} & \frac{3}{23} & \frac{1}{8} \end{array}\right] & p=\left[\begin{array}{c} 0.230\\ 0.648\\ 0.122 \end{array}\right] \end{array}$$

If $p={\left[w_{1}\cdots w_{n}\right]}^{T}$ are priorities of a set of alternatives, then, ideally, the comparison matrix M will have elements $m_{ij}=\frac{w_{i}}{w_{j}}$. In such a matrix, for any i,j,k∈{1,…,n}

$$m_{ij}\cdot m_{jk}=\frac{w_{i}}{w_{j}}\cdot \frac{w_{j}}{w_{k}}=\frac{w_{i}}{w_{k}}=m_{ik}$$

This property is called consistency. It can be shown that a consistent reciprocal matrix has rank 1, its largest eigenvalue is *n*, and it is the only eigenvalue not equal 0. All columns are eigenvectors. Since *j*-th column of M is equal $\frac{1}{w_{j}}\cdot p$, it follows that p is an eigenvector corresponding to the eigenvalue *n*, i.e., M·p=n·p. Small perturbations in elements of a comparison matrix lead to small perturbations in its primary eigenvector [38]. In practice, comparison matrix is always square positive and reciprocal, but it is usually not consistent. For small departures from consistency, the primary eigenvector is still a good approximation of priorities. Saaty [35] proposed two measures of consistency. The first measure, a consistency index CI, is based on the fact that a positive reciprocal square matrix M has a single largest eigenvalue $\lambda_{max}$ such that $\lambda_{max}\geq n$, and $\lambda_{max}=n$ if, and only if M is consistent [35]:

(2) $$CI=\frac{\lambda_{max}-n}{n-1}$$

The consistency index CI is 0 if, and only if M is consistent. Unfortunately, CI depends on the dimension of M, and no single cut-off value can be proposed as a criterion for significant inconsistency. In order to resolve this problem, Saaty [35] proposed to compare the value of consistency index to an average of consistency indices from a large number of random reciprocal matrices with values taken from the Saaty scale. For a positive reciprocal matrix M, a consistency ratio CR is defined as a ratio of its consistency index and an average of consistency indices of conformant random reciprocal matrices. Saaty [35] recommends accepting as reasonably consistent matrices with CR<0.1.

For example, for the matrix of pairwise comparisons M in expression (1), the largest eigenvalue is 3.0037. The matrix M is the result of pairwise comparisons among three criteria, thus n=3. From expression (2)

$$CI=\frac{3.0037-3}{3-1}=\frac{0.0037}{2}=0.0018$$

This value is compared to a reference value RI in [35]. For n=3, the reference value is RI=0.52, and

$$CR=\frac{CI}{RI}=\frac{0.00185}{0.52}=0.0036<0.1.$$

Since CR is much smaller than the recommended cut-off value of 0.1, we may conclude that the matrix M is consistent.

Indeed, if we use symbols $A_{1},A_{2},A_{3}$ for the alternatives that were compared, than $A_{2}$ is dominates $A_{1}$ by 3 (because $m_{21}=3$), and $A_{1}$ is dominates $A_{3}$ by 2 ($m_{13}=2$). If comparisons were consistent, we would expect $A_{2}$ to dominate $A_{3}$ by approximately 3×2=6. We have $m_{23}=5$. This difference is acceptable. If we were to change $m_{23}$ to 2, and $m_{32}$ to 0.5, saying then in fact $A_{2}$ dominates $A_{3}$ only by 2, for the new matrix the largest eigenvalue would be 3.1356, yielding CI=0.0678, and CR=0.1304>0.1, and the new matrix would be inconsistent.

A consistency ratio was computed for each expert’s pairwise comparison matrix, and for the group pairwise comparison matrices.

For all experts, this was the first time they participated in a multi-criteria decision-making with the AHP. The experts used SuperDecisions software to input results of their pairwise comparisons [39]. SuperDecisions software provides information on consistency ratio. Some experts did not provide consistent assessments at first. After additional explanations, these experts corrected their assessments. Moderators of the workshop did not comment on the expert’s assessments, they only explained the meaning of consistency, and which values of the consistency ratio are acceptable.

Once criteria weights were calculated, they were used to prioritize (rank) the hospitals. The selected indicators were normalized, using the following formula:

$${\hat{I}}_{hi}^{e}=\left\{\begin{array}{cc} \frac{I_{hi}^{e}-\min_{h}I_{hi}^{e}}{\delta_{i}^{e}} & \mathrm{if}\;\mathrm{larger}\;\mathrm{value}\;\mathrm{is}\;\mathrm{better}\\ \frac{\max_{h}I_{hi}^{e}-I_{hi}^{e}}{\delta_{i}^{e}} & \mathrm{if}\;\mathrm{smaller}\;\mathrm{value}\;\mathrm{is}\;\mathrm{better} \end{array}\right.$$

where $I_{hi}^{e}$ is value of the *i*-th indicator of entity *e* for hospital *h*, ${\hat{I}}_{hi}^{e}$ is its normalized value, and $\delta_{i}^{e}$ is the scaling factor for the *i*-th indicator for entity *e*. For the normalized indicators larger values indicate better performance. Value of a normalized indicator for the worst-performing hospital with respect to that indicator is 0. If difference between two hospitals on an indicator is equal to the criterion used in pairwise comparisons, then the normalized indicator of the better performing hospital is larger by 1.

Composite indicators were calculated as:

$$C_{h}^{e}=\sum_{i}w_{i}^{e}\cdot {\hat{I}}_{hi}^{e}$$

where $w_{i}^{e}$ is weight for the *i*-th criterion for entity *e*. Finally, for each entity, hospitals were ranked (prioritized) by the value of the respective composite indicator.

### 2.4. Sensitivity Analysis

To assess the impact of calculated weights on the hospital ranking, we performed a Monte Carlo experiment. For each entity, we made 100,000 replications of a simulation. In each replication, for each criterion and entity, we generated a random weight from the uniform distribution on the interval ±15% around the respective weight obtained through the AHP. For each hospital and entity, the value of the composite indicator was calculated using these weights, and hospitals were ranked. Variation in ranking was visualized using violin plots [40].

The SuperDecisions software and spreadsheet calculator were used for pairwise comparisons, aggregation of comparison matrices, estimation of weights and consistency ratios [39]. Normalization of indicators, calculation of composite indicators, and sensitivity analyses were done in R and RStudio [41,42].

## 3. Results

### 3.1. Indicator Weights

#### 3.1.1. Acute Myocardial Infarction (Ami)

It is not possible to directly compare indicators, because their relative importance depends on difference in values. Therefore, for each indicator, a criterion indicating effect size was defined (Table 3). The range of individual indicator values and the need to satisfy the homogeneity axiom (Axiom 2) guided the selection of the effect sizes. If the criteria did not satisfy the homogeneity axiom (i.e., were not 9-homogeneous), the experts would be unable to conduct pairwise comparisons using the Saaty scale.

Criteria in Table 3 were used for the pairwise comparisons. For each pair of indicators, a comparison question was formulated. For example, the experts were asked: “When ranking best-performing hospitals in Croatia with respect to the entity AMI, which criterion (1) decreasing the age and gender standardized AMI 30 day in-hospital (same hospital) mortality rate by 5%, or (2) decreasing the readmission rate for AMI within 30 days of discharge by 5%, is more important and by how much on the Saaty scale?”. A second variant of the question for each pairwise comparison was formulated as follows: “Two hospitals are almost equal respecting all indicators. They differ in only two indicators. Hospital 1 has age and gender standardized AMI 30 days in-hospital (same hospital) mortality rate 5% lower than Hospital 2. Hospital 2 has the readmission rate for AMI within 30 days of discharge 5% lower than Hospital 1. Which hospital is better and how much using the Saaty scale?”.

Nine AMI experts provided pairwise comparisons. Individual comparison matrices were aggregated into a group pairwise comparison matrix using the geometric mean (Table 4). All individual pairwise comparison matrices, as well as the aggregated matrix, were consistent.

Table 5 reports individual and group criteria weights. The group criteria weights were used for hospital rankings. Most experts thought that the most important indicator for AMI was the mortality rate, followed by the rate of prescription of aspirin and the readmission rate. Other indicators had more or less similar weights. Variability in weights was the most prominent for the mortality rate, and the rate of assessment of a comorbidity index. The experts S7 and S4 put much more importance than others on the length of stay. The expert S7 also put much less importance on the rate of prescribing an aspirin therapy. On the other hand, the expert S9 put much more importance than others on the rate of assessment of a comorbidity index. Since the geometric mean was used for aggregation of comparison matrices, individual extremes could not exert undue influence on the group comparison matrix.

#### 3.1.2. Cerebrovascular Insult (CVI)

Table 6 shows the list of criteria for the CVI indicators.

Number of pairwise comparisons per participant for criteria related to the CVI was 28. The pairwise comparison procedure was moderated, supplying questions about relative importance of criteria to ensure common understanding. Two examples of pairwise comparison questions for the CVI related criteria are: “When ranking the best-performed hospitals in Croatia with respect to the CVI, which criterion (1) decreasing the average length of hospital stay for stroke by 1 day or (2) decreasing the readmission rate for CVI within 30 days of discharge by 5%, is more important and how much on the Saaty scale?”, and “Two hospitals are almost equal in respect to all indicators. They differ in only two indicators. Hospital 1 has the average length of hospital stay for stroke 1 day shorter than Hospital 2. Hospital 2 has the readmission rate for CVI within 30 days of discharge 5% lower than Hospital 1. Which hospital is better and how much using the Saaty scale?”

There were 16 CVI experts who provided the judgments. Their comparison matrices were aggregated into a group pairwise comparison matrix using the geometric mean (Table 7). All individual pairwise comparison matrices, as well as the group comparison matrix, were consistent.

Most experts agreed that the most important indicator was the percentage of patients with CT scan or MRI done within the three hours of admission, followed by the mortality rate and the rate of prescribing the anticoagulant therapy. Other indicators were deemed to be of lower importance. It is interesting to note that the expert S13 clearly favored the mortality rate more than the others. The expert S18 assessed the percentage of patients released to a rehabilitation facility as more important than others, while the experts S15 and S16 clearly favored the percentage of records with admission time. The last two experts also had very similar estimates of all criteria weights. Variability among the experts’ weights was the highest for the mortality rate and the rate of prescribing the anticoagulant therapy. For other indicators, differences between the experts were not as pronounced.

Table 8 presents individual and the group criteria weights. The group criteria weights were used for hospital rankings.

#### 3.1.3. Antimicrobial Prophylaxis in Colorectal Surgery (Apc)

Table 9 lists criteria derived from indicators related to the APC.

Number of pairwise comparisons per participant for criteria related to the APC is 10. The pairwise comparison procedure was moderated, providing questions to ensure understanding. Examples of the used pairwise comparison questions are: “When ranking best-performing hospitals in Croatia with respect to the entity APC, which criterion (1) increasing a percentage of patients with the type of antibiotic prescribed compliant with the guidelines by 5% or (2) increasing a percentage of patients with the dose of antibiotic prescribed compliant with the guidelines by 5%, is more important and how much on the Saaty scale?”, and “Two hospitals are almost equal with respect to all indicators. They differ in only two indicators. In Hospital 1 the percentage of patients with the type of antibiotics prescribed compliant with the guidelines is 5% higher than in Hospital 2. In Hospital 2 the percentage of patients with a dose of antibiotics prescribed compliant with the guideline 5% higher in than Hospital 1. Which hospital is better and how much using the Saaty scale?”.

Eleven experts for the APC provided judgments. Eleven pairwise comparison tables were aggregated into a group pairwise comparison table using the geometric mean (Table 10). All individual pairwise comparison tables were consistent. Additionally, the group pairwise comparison table was consistent.

Table 11 contains the individual and the group criteria weights for the APC. The group criteria weights were used for hospital ranking. According to the group weights, the most important indicator is the time of initial prophylaxis, followed by the drug type, and the dose. The APC was the entity with the highest variability of individual experts’ weights. However, the APC was also the only entity for which there was a significant correlation between some indicators, thus variation in weights has the lowest impact. This was also the only entity for which all indicators were indicators of process (compliance with the guidelines). Variability between the experts’ weights was the largest for the type of antibiotic, followed by the time of initial administration. The expert S30’s weight for the start of the prophylaxis was the highest, and diverged the most from the other experts’ weights. The same can be said for the expert S32 and the timing of the end of prophylaxis.

**Figure 5:**
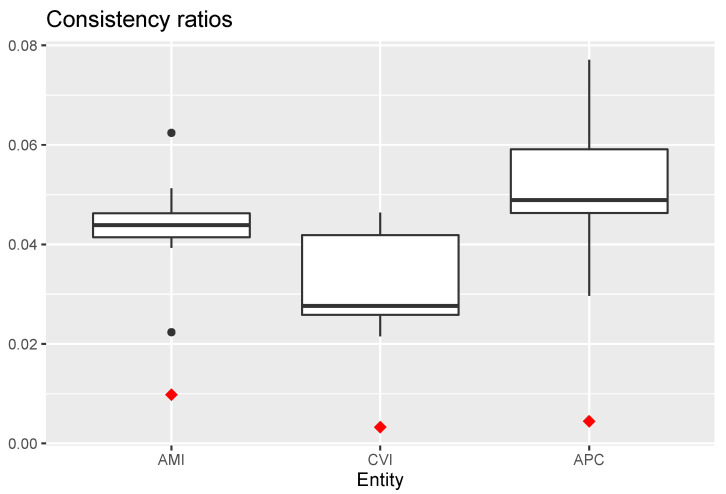
Boxplots of consistency ratio values for the three entities. Red diamonds indicate the value of consistency ratio for the group comparison matrices. AMI = acute myocardial infarction; CVI = cerebrovascular insult; APC = antimicrobial prophylaxis in colorectal surgery.

Figure 5 shows boxplots of consistency ratios for the three entities. Red diamonds indicate consistency ratios for the aggregated group comparison matrices. Consistency ratios for CVI were the lowest (the best), followed by those for AMI. Consistency ratios for APC were the highest, but still well below the recommended threshold of 0.1. Consistency ratios for the aggregated group comparison matrices were lower than those of the individual expert’s comparison matrices.

### 3.2. Sensitivity Analysis

**Figure 6:**
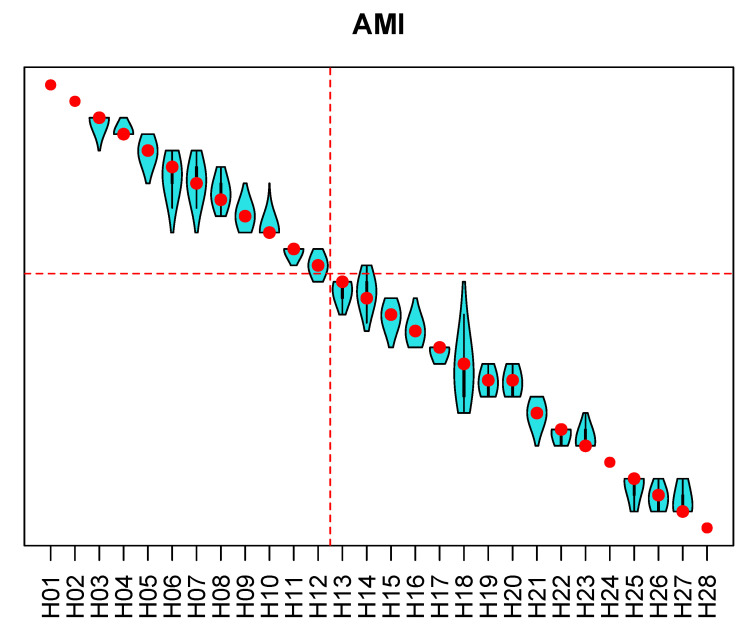
Results of the sensitivity analysis for the ranking of hospitals with respect to the acute myocardial infarction. Violin plots show distribution of ranks from the Monte Carlo simulation. Dashed red lines indicate the 40% best-performing hospitals.

**Figure 7:**
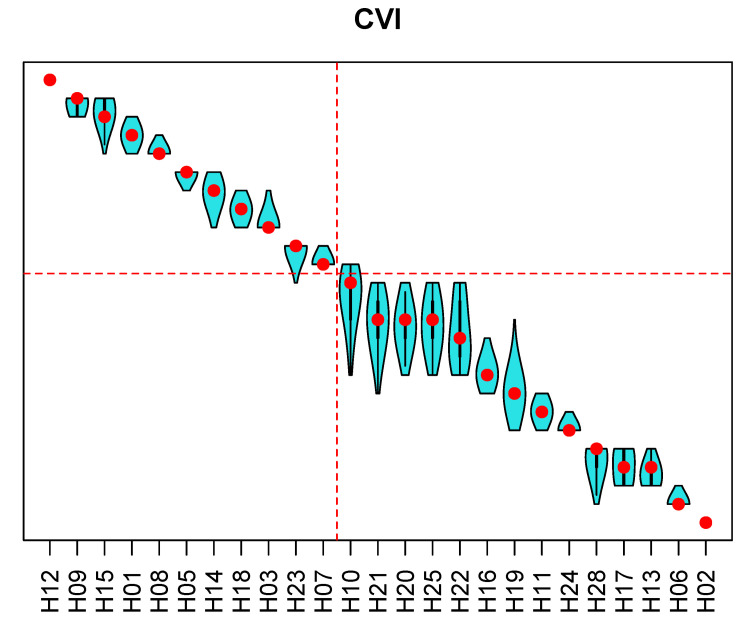
Results of the sensitivity analysis for the ranking of hospitals with respect to the cerebrovascular insult. Violin plots show distribution of ranks from the Monte Carlo simulation. Dashed red lines indicate the 40% best-performing hospitals. The same numbering of hospitals is used as in Figure 6.

**Figure 8:**
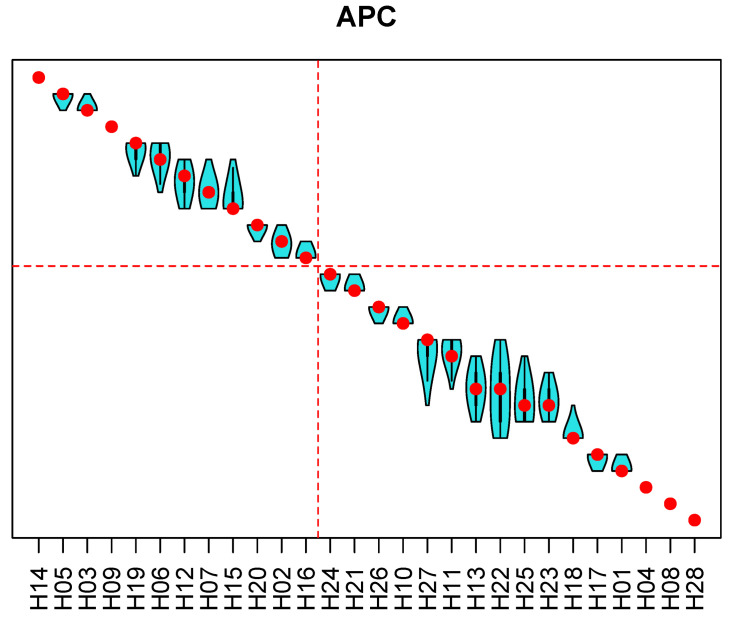
Results of the sensitivity analysis for the ranking of hospitals with respect to the antimicrobial prophylaxis in colorectal surgery. Violin plots show distribution of ranks from the Monte Carlo simulation. Dashed red lines indicate the 40% best-performing hospitals. The same numbering of hospitals is used as in Figure 6.

Results of the sensitivity analysis for the rankings with respect to the AMI, the CVI and the APC are presented in Figure 6, Figure 7 and Figure 8. In the figures, the hospitals are ordered from the best ranking on the left to the worst ranking on the right. Red points represent a hospital rank (from top to bottom), and the violin plots show distributions of ranks across 100,000 replications of the Monte Carlo simulation experiment. For all three entities, the top-performing and the worst-performing hospitals do not show ranking reversals. For most of the hospitals, the rank variation spans two to three ranks. Wider spans are present among the worst-performing hospitals. The group of the top 40% hospitals is generally stable for all three entities, and the proposed methodology enabled achieving the goal of selecting the 40% best-performing hospitals.

### 3.3. Communication

**Figure 9:**
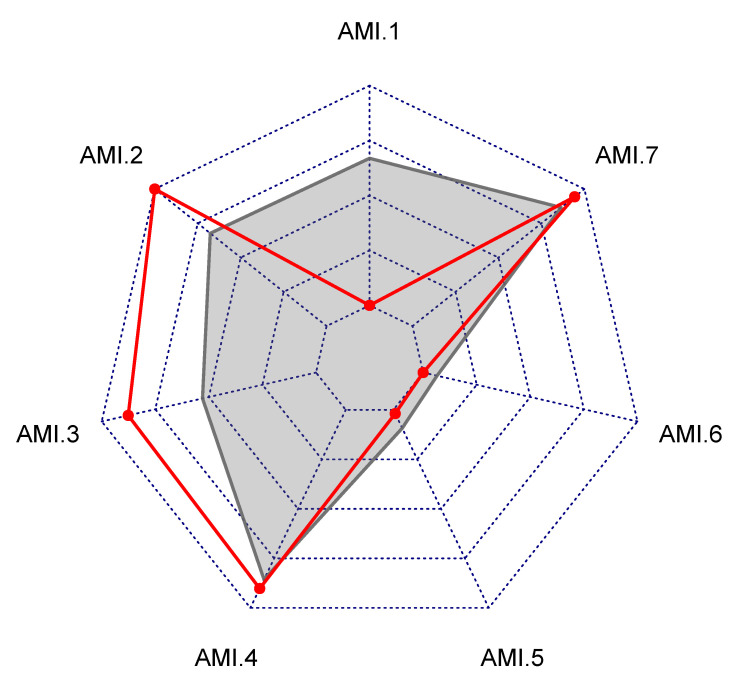
An example of a radial plot showing average values of indicators for AMI as the outer contour of the gray area, and values for a chosen hospital as a red contour. Values of each indicator range from the worst value in the center, to the best value at the rim.

Public report on hospital rankings displayed violin plots, such as those in Figure 6, Figure 7 and Figure 8, showing only names of the hospitals that were among the 40% best performing (to the left of the red line). Each audited hospital also received an individual report, indicating hospital’s position in the violin plots. Additionally, the individual report contained a radial plot for each entity, showing values of indicators for the individual hospital, and the average values of indicators for all ranked hospitals. An example of a radial plot is shown in Figure 9. Values of each indicator range between the value reflecting the worst performance in the center and the value reflecting the best performance at the rim. In the example, values of indicators AMI.2 and AMI.3 reflect better than average performance. Values of AMI.4 and AMI.7 are slightly better than the average. Values of AMI.1, AMI.5, and AMI.6 reflect the worst performance. Those are indications of areas where there is a room for improvement.

## 4. Discussion

In 2017 Schiele et al. [43] published a position paper of the Acute Cardiovascular Care Association on quality indicators for acute myocardial infarction. Their recommendations include, among others, indicators we use in the present study—routine measurement of relevant times for the reperfusion process, low dose aspirin therapy prescribed, assessment of risk index, and 30-day standardized mortality rate. Our individual indicators also comprise readmission rate, average length of stay, and percentage of patients discharged to a rehabilitation facility.

A systematic analysis on stroke quality metrics is provided by Parker et al. [12], who conclude that outcome indicators may not reflect accurately quality of healthcare, and that process measures should remain the first choice when comparing hospitals. Nishimura et al. [44] develop quality indicators for stroke centers in Japan. Among others, they recommend measurement of time of admission and time between arrival and CT or MRI scan, anticoagulant therapy, and assessment of severity, as used in this study. Our individual indicators also comprise readmission rate, average length of stay, 30-day standardized mortality, and percentage of patients discharged to a rehabilitation facility.

Schmitt et al. [45] report on a multi-center study of surgical antibiotic prophylaxis. They analyze indication, dose, drug type, initial time of antibiotic prophylaxis, and duration of prophylaxis. The same indicators, represented as percentage of patients treated compliant to the national guidelines, were used in this study.

Hospital rankings have been designed with different goals, different domains, sources, and types of data, and with different methods. Dong et al. [46] provide an overview of ranking systems in China and their goals, which include providing guidance and information to patients, measure scientific output and reputation, measure competitiveness, and measure performance. Sources of data used for hospital rankings include e.g., patient surveys, administrative databases, public reports, medical records, expert assessments, research citation databases, and self-reporting [46,47,48]. Mortality, compliance with standard procedures, length of stay, readmission, number of beds and patients, number and specialty of personnel, participation in clinical trials, timeliness, patient experience, social reputation, and many other indicators have been used for hospital ranking (e.g., [46,47,48,49]).

Our approach to designing a composite hospital performance indicator focused on a weighted average of normalized individual indicators chosen based on national guidelines and the availability of relevant data. The goal of our ranking was to identify top-performing hospitals, and the sources of data were public reports based on self-reporting, administrative databases, medical records scanned during the audit, and the experts assessment. The individual indicators were indicators of outcomes (e.g., mortality), processes (e.g., time of administration of antimicrobial prophylaxis), and efficiency (e.g., length of stay). To ensure acceptance of the ranking, we decided to use participatory (group) multi-criteria decision-making to choose the weighting scheme. Experts from the audited hospitals provided pairwise comparisons between the chosen criteria, and the resulting pairwise comparison matrices were highly consistent. According to Jacobs, Goddard and Smith [9] composite indicators are easy to interpret, enable comparisons between hospitals, and provide information for regulatory actions and hospital users. They warn that it is necessary to apply risk adjustments on indicators that may be influenced by case-mix or other sources of extra variability, and to perform uncertainty and sensitivity analysis. We have done both—the age and gender standardization, and sensitivity analysis. In our sensitivity analysis, similar to Jacobs, Goddard and Smith simulation [9], variability of ranking was higher for hospitals around the median, and ranking of hospitals in the upper and the lower quartiles was less variable.

Dey and Harihara [50] have used the AHP for hospital performance comparison. They find many advantages in using the AHP as a multi-criteria decision-making tool for hospital performance measurement, for example, possibility to include many different criteria and encompass multi-factorial nature of healthcare service, implementation of a group decision-making process, and the AHP’s sound mathematical basis. On the other hand, choice of the measurement scale for criteria and aggregation over levels of hierarchy were seen as the AHP’s shortcomings. Dey and Harihara [50] rate criteria on a three-point scale low/poor, average, and high/good with weights of 0.1, 0.3, and 0.6, respectively. We use quantitative individual indicators as criteria, and the AHP weights are used for aggregation into a composite indicator, which reduces the significance of these shortcomings.

Many researchers combine successfully the AHP with a wide range of different methods for evaluating hospital performance. Examples include Ulkhaq et al. [47] who combine the AHP for determining the weights of criteria and subcriteria, and the technique for order preference by similarity to ideal solution (TOPSIS) to find the best alternative in terms of service quality. Their approach is similar to ours in the way they use the AHP for structuring and weighting the criteria used for hospital ranking, but then choose another method for the final ranking of the hospitals. In the AHP, hierarchical structuring of the criteria can reduce the number of pairwise comparisons between the criteria; however, all alternatives (i.e., hospitals) still must be compared in pairs regarding each criterion at the level above the alternatives. The TOPSIS used by Ulkhaq et al. [47], and the composite indicators approach that we use, eliminate the need for pairwise comparisons between the hospitals. Without this step, the method would not be scalable to many hospitals. With the composite indicator approach that we use it is easier to interpret contributions of individual indicators to the overall score. In TOPSIS, scores are distances in a multidimensional space, and it is not easy to interpret contribution of individual indicators to the overall score and the rank.

Sakti, Sungkono, and Sarno [51] combine the AHP with a multi-objective optimization approach based on ratio analysis (MOORA) and then average the rankings obtained by these two methods. They use the AHP for criteria prioritization in both methods, and then do both the AHP comparisons, and the MOORA ranking for the alternatives. With only six criteria and 10 hospitals, they need 270 pairwise comparisons between hospitals regarding the criteria (the last level of the hierarchy). This approach is not scalable to a much larger number of hospitals. On the other hand, use of the AHP only for criteria weighting, and the MOORA for the final ranking would be scalable. The MOORA score is similar to the composite indicator score, because both scores are computed as a weighted sum of standardized individual criteria values. However, the MOORA, and the previously mentioned TOPSIS, use a simple standardization that is applicable to scores that are measured on the same scale, such as those obtained in surveys. With criteria measured on different scales, the scaling factors must be chosen with the goal of maintaining 9-homogeneity of the compared criteria, and they must be communicated to the experts who participate in the pairwise comparisons. Thus, neither the MOORA, nor the TOPSIS could be used for ranking hospitals with indicators used in our research.

Our research is based on the implementation of the AHP method in combination with computing of composite indicators, which best fits the observed problem. One of the strong aspects of this research were the experts who participated in the research. All hospitals were invited to participate in the process, and most of them took advantage of this opportunity, since the final rankings have a huge impact on hospitals’ reputation, and indirectly also on the state funding. The facts that only names of the top-performing hospitals were publicly declared, that sensitivity to weights was acknowledged, and that experts from the audited hospitals were involved in decision-making, probably contributed to good acceptance of the ranking. We did not receive any criticism from the audited hospitals.

The fact that hospitals also received individual reports with indication of their rank with respect to each entity, and a breakdown of individual indicators that contributed to their results, facilitated concrete action on improving performance of individual hospitals. It was also interesting to identify hospitals whose rank was highly dependent on the choice of weights (i.e., those which had long violin plots), as well as those whose rankings on the three entities differed significantly. Those hospitals show uneven quality of clinical and management practices, and their good rank in respect to one entity may be a result of a small team working in one specialty, and not the consistent quality management practices at the hospital level. Our communication strategy was to give praise to the best, while providing individualized actionable information to all. Such communication strategy is the key to translating results of this research into clinical practice.

Limitation of this research include:

**Small documentation sample during the audit.** We selected a simple random sample of patients for each entity. However, with only 50 patients per entity, estimates of rates have large standard errors, and contribute to the uncertainty of rankings. Sample size was limited by the resources available for performing the audit. Indicators of standardized mortality and average length of stay were collected from the records of the AQAH and CHIF, and were based on all patients in the target year.

**Data quality and availability.** There were discrepancies in data collecting procedures that made data from different hospitals incomparable. Some hospitals did not record all information necessary for computing the selected indicators. Thus, the initial selection of potential indicators for the audit was reduced to a smaller number of criteria for ranking. We could only use indicators that could be computed for all hospitals, and that were comparable. Since inadequate data collection is also a sign of poor-quality management, in lieu of targeted indicators, we introduced indicators of data availability.

**Potentially biased weighting.** Participation of experts from the audited hospitals had a beneficial impact on the acceptance of the ranking. Their deep understanding of the clinical and data collection practices in the audited hospitals could also have influenced the pairwise comparisons, by eliciting lower importance assessments for indicators based on low quality data (thus also reducing the impact of low data quality). On the other hand, the experts may have been aware of their hospital’s strengths, and could have assessed the indicators related to these strengths as having a higher importance, thus introducing a bias. This may also be one of the reasons for variability in weights between the experts. However, since all experts’ pairwise comparisons contributed the same to the group comparison matrix, such biased individual assessments would have compensatory effect.

## 5. Conclusions

The AHP method is a versatile multi-criteria decision-making method, which has been widely applied in healthcare decision-making. In practice, the AHP was successfully combined with a wide range of approaches, including TOPSIS, MOORA, and DEA. We demonstrate that the AHP can also be used to design composite indicators for ranking hospitals based on their performance and service quality. Group decision making, supported by the AHP, takes advantage of professionals’ knowledge, and helps establish trust through participatory decision making.

We have achieved our research goals:

1. We presented a methodology for ranking top-performing hospitals at the national level, which involves experts from the field, and aggregates their possibly conflicting opinions. The methodology is based on the commonly used method—the AHP. It supports important aspects of the hospital ranking problem:
  - It enables modeling complex decision-making structures appearing in the hospital ranking problem, using a hierarchy of criteria on as many levels as necessary. The problem can be structured in a way that optimizes the number of inputs required from the experts.
  - It facilitates aggregation of different opinions into a common compromise decision.
  - Contribution of individual indicators to the overall score is easy to understand, and that enables translation of the results in the clinical practice.
2. The methodology was successfully applied in the case of Croatian public acute hospitals.
  - A hierarchical decision-making structure of the hospital ranking problem was created, using evidence-based hospital quality, safety, and performance indicators, respecting availability of data from the audit, and the Croatian national health information systems.
  - Experts for the AMI, the CVI and the APC from the audited hospitals provided input (pairwise comparisons).
  - Combining hospital indicators with the AHP-based weights into composite indicators enabled ranking of the 40% top-performing hospitals at the national level. Even though rank reversal was present in sensitivity analysis, the best and the worst ranking hospitals did not show rank reversals. Additionally, the sensitivity analysis confirmed that the group of the 40% top-performing hospitals was stable. For hospitals ranking around median and lower, ranges of ranks from sensitivity analysis were wider.

Possible venues of future research include looking into:

**Criteria prioritization:** it would be interesting to explore and compare how well other multi-criteria decision-making methods, for instance methods that take into account dependencies among the criteria (e.g., the analytic network process, ANP [52], the decision-making trial and evaluation laboratory, DEMATEL [53], or the social network analysis process, SNAP [54]), solve the hospital ranking problem. Specifically, it would be interesting to analyze whether methods with higher complexity achieve higher stability of rankings.

**Experts’ input:** further analysis of the individual expert’s comparison matrices and priorities might provide additional insight into, e.g., how individual experts influence the group priorities, is there an association between expert priorities and their respective hospital’s indicators or rankings, and whether clinical experts perceive outcome or process indicators as more important measures of hospital quality.

## Figures and Tables

**Table 1:** Indicators and sources of data by clinical entity.

Entity	Indicator (Abbreviation)	Source ${}^{1}$
AMI ${}^{2}$	Age and gender standardized AMI 30 days in-hospital (same hospital) mortality rate (mort-30-ami)	AQAH
	Readmission rate for AMI within 30 days of discharge (readmission-30-ami)	CHIF
	Average length of hospital stay for AMI (alos-ami)	CHIF
	Percentage of AMI patients with aspirin therapy prescribed at discharge (%aspirin-ther-ami)	audit
	Percentage of AMI patients with comorbidity index assessed (%comorb-ix-ami)	audit
	Percentage of AMI patients discharged from the hospital to a rehabilitation facility (%rehabilitation-ami)	audit
	Percentage of AMI patients with admission time recorded in the medical record (%admission-time-ami)	audit
CVI ${}^{3}$	Age and gender standardized CVI 30 days in-hospital (same hospital) mortality rate (mort-30-cvi)	AQAH
	Readmission rate for CVI within 30 days of discharge (readmission-30-cvi)	CHIF
	Average length of hospital stay for CVI (alos-cvi)	CHIF
	Percentage of CVI patients with anticoagulant therapy administrated (%anticoag-ther-cvi)	audit
	Percentage of CVI patients with CT scan or MRI done within 3 h of admission (%CT-MRI-cvi)	audit
	Percentage of CVI patients with clinical state index assessed (%clinical-ix-cvi)	audit
	Percentage of CVI patients discharged from the hospital to a rehabilitation facility (%rehabilitation-cvi)	audit
	Percentage of CVI patients with admission time recorded in the medical record (%admission-time-cvi)	audit
APC ${}^{4}$	Percentage of patients with antibiotic prescribed respecting the national guidelines (%antibiotic-apc)	audit
	Percentage of patients with a dose of antibiotics prescribed respecting the national guidelines (%dose-apc)	audit
	Percentage of patients with antibiotic administered respecting the national guidelines (%apply-apc)	audit
	Percentage of patients with antibiotic therapy started respecting the national guidelines (%start-apc)	audit
	Percentage of patients with antibiotic therapy ended respecting the national guidelines (%end-apc)	audit

${}^{1}$ Source of data: CHIF = Information system of the Croatian Health Insurance Fund; AQAH = Reports of the Agency for Quality and Accreditation in Health and Social Care. ${}^{2}$ Acute myocardial infarction. ${}^{3}$ Cerebrovascular insult. ${}^{4}$ Antimicrobial prophylaxis in colorectal surgery.

**Table 2:** Saaty’s fundamental scale.

Importance	Definition
1	Equal importance
3	Moderate importance
5	Strong importance
7	Very strong (or demonstrated) importance
9	Extreme importance
2, 4, 6, 8	Intermediate values
Reciprocals of 1–9	If activity *i* has one of the above nonzero numbers assigned to it when compared with activity *j*, then *j* has the reciprocal value when compared with *i*

**Table 3:** Indicators and criteria for the acute myocardial infarction (AMI).

Abbreviation	Indicator	Criterion
mort-30-ami	Age and gender standardized 30 days in-hospital (same hospital) AMI mortality rate	Decreasing the age and gender standardized 30 days in-hospital (same hospital) AMI mortality rate by 5%
readmission-30-ami	Readmission rate for AMI within 30 days of discharge	Decreasing the readmission rate for AMI within 30 days of discharge by 5%
alos-ami	Average length of hospital stay for AMI	Decreasing the average length of hospital stay for AMI by 1 day
%aspirin-ther-ami	Percentage of AMI patients with aspirin therapy prescribed at discharge	Increasing the percentage of AMI patients with aspirin therapy prescribed at discharge by 5%
%comorb-ix-ami	Percentage of AMI patients with comorbidity index assessed	Increasing the percentage of AMI patients with comorbidity index assessed by 5%
%rehabilitation-ami	Percentage of AMI patients discharged from the hospital to a rehabilitation facility	Increasing the percentage of AMI patients discharged from the hospital to a rehabilitation facility by 5%
%admission-time-ami	Percentage of AMI patients with admission time recorded in the medical record	Increasing the percentage of AMI patients with admission time recorded in the medical record by 5%

**Table 4:** Group pairwise comparison matrix for the acute myocardial infarction (AMI).

	C1	C2	C3	C4	C5	C6	C7
C1. mort-30-ami	1.000	3.465	3.289	1.397	3.038	4.189	3.067
C2. readmission-30-ami	0.289	1.000	3.121	0.550	2.209	1.917	1.901
C3. alos-ami	0.304	0.320	1.000	0.295	0.856	0.768	0.765
C4. %aspirin-ther-ami	0.716	1.817	3.388	1.000	2.300	4.413	2.648
C5. %comorb-ix-ami	0.329	0.453	1.168	0.435	1.000	1.196	0.630
C6. %rehabilitation-ami	0.239	0.522	1.303	0.227	0.836	1.000	1.041
C7. %admission-time-ami	0.326	0.526	1.307	0.378	1.587	0.960	1.000

**Table 5:** AMI criteria weights based on individual comparison matrices, and the group criteria weights. (S1 to S9 indicate experts participating in the AHP exercise).

	Criteria						
Experts	mort-30-ami	readmission-30-ami	alos-ami	%aspirin-ther-ami	%comorb-ix-ami	%rehabilitation-ami	%admission-time-ami
S1	0.360	0.110	0.036	0.226	0.056	0.149	0.063
S2	0.261	0.124	0.075	0.329	0.041	0.131	0.039
S3	0.237	0.196	0.039	0.273	0.113	0.056	0.087
S4	0.344	0.229	0.130	0.173	0.041	0.028	0.056
S5	0.352	0.174	0.042	0.239	0.056	0.029	0.108
S6	0.410	0.161	0.059	0.209	0.032	0.087	0.042
S7	0.225	0.095	0.255	0.057	0.117	0.053	0.198
S8	0.354	0.113	0.040	0.222	0.057	0.151	0.063
S9	0.080	0.055	0.024	0.286	0.354	0.038	0.163
Group 1	0.307	0.148	0.066	0.237	0.080	0.073	0.090

**Table 6:** Indicators and criteria for the cerebrovascular insult (CVI).

Abbreviation	Indicator	Criterion
mort-30-cvi	Age and gender standardized 30 days in-hospital (same hospital) CVI mortality rate	Decreasing the age and gender standardized 30 days in-hospital (same hospital) CVI mortality rate by 5%
readmission-30-cvi	Readmission rate for CVI within 30 days of discharge	Decreasing the readmission rate for CVI within 30 days of discharge by 5%
alos-cvi	Average length of hospital stay for CVI	Decreasing the average length of hospital stay for CVI by 1 day
%anticoag-ther-cvi	Percentage of CVI patients with anticoagulant therapy administrated	Increasing the percentage of CVI patients with anticoagulant therapy administrated by 5%
%CT-MRI-cvi	Percentage of CVI patients with CT scan or MRI done within 3 h of admission	Increasing the percentage of CVI patients with CT scan or MRI done within 3 h of admission by 5%
%clinical-ix-cvi	Percentage of CVI patients with clinical state index assessed	Increasing the percentage of CVI patients with clinical state index assessed by 5%
%rehabilitation-cvi	Percentage of CVI patients discharged from the hospital to a rehabilitation facility	Increasing the percentage of CVI patients discharged from the hospital to a rehabilitation facility by 5%
%admission-time-cvi	Percentage of CVI patients with admission time recorded in the medical record	Increasing the percentage of CVI patients with admission time recorded in the medical record by 5%

**Table 7:** Group pairwise comparison matrix for the cerebrovascular insult (CVI).

	C1	C2	C3	C4	C5	C6	C7	C8
C1. mort-30-cvi	1.000	2.884	2.904	1.380	0.993	2.266	2.299	2.166
C2. readmission-30-cvi	0.347	1.000	0.959	0.670	0.326	1.106	1.025	1.376
C3. alos-cvi	0.344	1.042	1.000	0.555	0.251	1.225	1.243	1.389
C4. %anticoag-ther-cvi	0.724	1.492	1.803	1.000	0.575	2.172	1.492	1.670
C5. CT-MRI-cvi	1.052	3.100	4.084	1.740	1.000	3.379	3.800	3.296
C6. %clinical-ix-cvi	0.441	0.904	0.817	0.460	0.304	1.000	0.898	0.846
C7. %rehabilitation-cvi	0.435	0.976	0.805	0.670	0.275	1.113	1.000	1.299
C8. %admission-time-cvi	0.462	0.727	0.720	0.599	0.303	1.355	0.770	1.000

**Table 8:** CVI criteria weights based on individual comparison matrices, and the group criteria weights. (S1 to S9 indicate experts participating in the AHP exercise).

	Criteria							
Experts	mort-30-cvi	readmission-30-cvi	alos-cvi	%anticoag-ther-cvi	CT-MRI-cvi	%clinical-ix-cvi	%rehabilitation-cvi	%admission-time-cvi
S10	0.214	0.035	0.054	0.218	0.131	0.092	0.148	0.108
S11	0.271	0.162	0.088	0.036	0.246	0.081	0.070	0.045
S12	0.185	0.040	0.044	0.166	0.273	0.058	0.101	0.133
S13	0.433	0.137	0.033	0.129	0.144	0.032	0.045	0.046
S14	0.142	0.053	0.099	0.339	0.221	0.027	0.075	0.044
S15	0.113	0.043	0.076	0.027	0.322	0.145	0.050	0.224
S16	0.115	0.043	0.077	0.031	0.317	0.136	0.058	0.224
S17	0.237	0.055	0.092	0.240	0.240	0.074	0.038	0.024
S18	0.118	0.045	0.083	0.099	0.178	0.160	0.226	0.089
S19	0.052	0.059	0.104	0.316	0.266	0.067	0.095	0.039
S20	0.283	0.130	0.094	0.051	0.324	0.023	0.056	0.040
S21	0.295	0.106	0.099	0.245	0.121	0.041	0.029	0.064
S22	0.316	0.194	0.037	0.135	0.184	0.024	0.077	0.032
S23	0.211	0.051	0.117	0.228	0.235	0.061	0.051	0.046
S24	0.183	0.147	0.024	0.113	0.234	0.153	0.086	0.060
S25	0.081	0.066	0.149	0.151	0.351	0.040	0.087	0.075
Group 2	0.203	0.084	0.084	0.138	0.262	0.071	0.082	0.076

**Table 9:** Indicators and criteria for the antimicrobial prophylaxis in colorectal surgery (APC).

Abbreviation	Indicator	Criterion
%antibiotic-apc	Percentage of patients with antibiotic prescribed respecting the guidelines	Increasing the percentage of patients with antibiotic prescribed respecting the guidelines by 5%
%dose-apc	Percentage of patients with a dose of antibiotics prescribed respecting the guidelines	Increasing the percentage of patients with a dose of antibiotics prescribed respecting the guidelines by 5%
%apply-apc	Percentage of patients with antibiotic administered respecting the guidelines	Increasing the percentage of patients with antibiotic administered respecting the guidelines by 5%
%start-apc	Percentage of patients with antibiotic therapy start respecting the guidelines	Increasing the percentage of patients with antibiotic therapy start respecting the guidelines by 5%
%end-apc	Percentage of patients with antibiotic therapy end respecting the guidelines	Increasing the percentage of patients with antibiotic therapy end respecting the guidelines by 5%

**Table 10:** Group pairwise comparison matrix for the antimicrobial prophylaxis in colorectal surgery (APC).

	C1.	C2.	C3.	C4.	C5.
C1. %antibiotic-apc	1.000	1.562	2.798	0.808	2.771
C2. %dose-apc	0.640	1.000	2.280	0.741	2.704
C3. %apply-ap	0.357	0.439	1.000	0.390	1.406
C4. %start-ap	1.238	1.349	2.564	1.000	3.249
C5. %end-apc	0.361	0.370	0.711	0.308	1.000

**Table 11:** APC criteria weights based on the individual comparison matrices, and the group criteria weights. (S1 to S9 indicate experts participating in the AHP exercise).

	Criteria				
Experts	%antibiotic-apc	%dose-apc	%apply-ap	%start-ap	%end-apc
S26	0.347	0.255	0.148	0.216	0.034
S27	0.336	0.201	0.177	0.252	0.034
S28	0.232	0.286	0.054	0.232	0.196
S29	0.492	0.110	0.060	0.306	0.032
S30	0.072	0.162	0.040	0.564	0.162
S31	0.172	0.111	0.089	0.414	0.214
S32	0.164	0.089	0.049	0.285	0.412
S33	0.245	0.365	0.234	0.118	0.037
S34	0.107	0.166	0.258	0.400	0.069
S35	0.452	0.279	0.052	0.187	0.030
S36	0.467	0.253	0.087	0.148	0.045
Group 3	0.282	0.221	0.109	0.301	0.088

## Data Availability

Data available under request to the authors.

## Abbreviations

AHP	analytic hierarchy process
AMI	acute myocardial infarction
ANP	analytic network process
APC	antimicrobial prophylaxis in colorectal surgery
AQAH	Agency for Quality and Accreditation in Health and Social Care
CHIF	Croatian Health Insurance Fund
CVI	cerebrovascular insult
DEA	data envelope analysis
DEMATEL	decision-making trial and evaluation laboratory
MCDM	multi-criteria decision-making
MOORA	multi-objective optimization method on the base of ratio analysis
PATH	performance assessment tool for quality improvement in hospitals
PrOACT	Problem formulation, Objectives, Alternatives, Consequences, Trade-offs, Uncertainties,
	Risk attitude, and Linked decisions
SAW	simple additive weighting
SNAP	social network analysis process
TOPSIS	technique for order preference by similarity to ideal solution
WHO	World Health Organization
